# Occupational Exposure to Second-Hand Tobacco Smoke: Development of a Job Exposure Matrix

**DOI:** 10.1093/annweh/wxab019

**Published:** 2021-04-03

**Authors:** Ruaraidh Dobson, Evangelia Demou, Sean Semple

**Affiliations:** 1 Institute for Social Marketing and Health, University of Stirling, Stirling, UK; 2 MRC/CSO Social and Public Health Sciences Unit, University of Glasgow, Berkeley Square, Glasgow, UK

**Keywords:** aerosols, exposure assessment, exposure estimation, occupational groups, smoking

## Abstract

Exposure to second-hand tobacco smoke (SHS) in the workplace has been largely controlled in most workplaces in many countries that have adopted smoke-free laws and regulations. Workers in offices, bars, restaurants, and many other settings have experienced substantial reductions in the frequency and intensity of their exposure to SHS. While current exposure to SHS of most non-smoking adults arises from living with a smoker there are likely to be some jobs where non-negligible exposure to SHS continues to occur. This study describes the development of a simple job exposure matrix (JEM) for SHS exposure for the UK working population in 2020 and identifies that at least 1.04 million workers are likely to be exposed to SHS while performing their job. Occupations with the highest frequency and intensity of exposure include those where workers carry out work tasks in private, domestic settings: including care workers and home carers. This SHS-JEM provides a novel method for assessing occupational exposure to SHS in other countries, and can act as a tool to identify priorities for policies to protect those workers who continue to be at risk from SHS.

**What’s important about this paper**

Second-hand smoke is a significant source of health harm in the workplace. This study developed a job exposure matrix (JEM) for this hazard, and found that more than a million people in the UK are likely exposed to second-hand smoke at work. The most impacted groups are worker that must work inside others’ homes, including care workers. The JEM developed in this work shows that, despite the UK’s comprehensive smoke-free public places laws, many workers may still be occupationally exposed to second-hand smoke.

## Introduction

Exposure to second-hand tobacco smoke (SHS) is a significant cause of adult ill-health, resulting in a range of cardiovascular and pulmonary illnesses (US Surgeon General, 2006). Exposure to SHS at work was previously estimated to be associated with the premature death of over 600 workers in the UK each year (Jamrozik, 2005).

Occupational exposure to SHS, once extremely common, has declined in many countries since the adoption of Article 8 of the WHO Framework Convention on Tobacco Control (World Health Organisation, 2003) which required smoke-free laws to restrict smoking in indoor public and workspaces (Angus and Semple, 2019). However, evidence from Scotland in 2016 suggests that nearly one in five non-smoking adults show evidence of exposure to SHS in the form of measurable levels of cotinine in their saliva. While the majority of these non-smokers encounter SHS at home from living with a smoker, it is likely that some of these non-smoking adults experience exposure to SHS within work settings. A recent review of international literature identified considerable qualitative data indicating that groups such as home care workers identify SHS as a key concern (Angus and Semple, 2019). Efforts to further reduce occupational SHS exposure have focussed on indoor environments where smoking continued to be permitted after the introduction of smoke-free public places legislation. This has included prisons, which are now smoke-free in Scotland, England, and Wales (Jayes *et al.*, 2019; Demou *et al.*, 2020).

A job exposure matrix (JEM) allows the estimation of individual exposure to a hazard (whether physical, like a pollutant, or psychological) by occupation. This can allow evidence-based policy decisions to be made, or retrospective exposures to be estimated based on occupation for the purposes of case–control studies, and can play an important role in understanding the relationship between past exposure and disease. A JEM can also be useful in identifying where future policy and control measures should be best targeted to further reduce exposure to occupational hazards. JEMs can be based on empirical evidence, expert judgement, or a combination of these two approaches.

JEMs have been developed for many occupational exposures, including ultraviolet light (Boiano *et al.*, 2020), asbestos (Scarselli *et al.*, 2020), and diesel exhaust particles (Plato *et al.*, 2020). However, we are unaware of any systematic JEM principally focussed on defining either past or current exposure to SHS by occupation, with only the Finnish Information System on Occupational Exposure (FINJEM) including occupational exposure to harmful constituents of SHS, such as polycyclic aromatic hydrocarbons (Kauppinen *et al.*, 2014). In this paper, we develop a preliminary SHS-JEM for the UK in 2020 to be validated in future research. We also consider how this could be developed for other countries and for past exposures.

## Methods

### Occupation information

Standard Occupational Classification (SOC) 2020 codes, developed by the UK Office of National Statistics (Office for National Statistics, 2020), were used to develop the JEM. These 412 four-digit codes include all classes of occupation in the UK. The 26 two-digit SOC codes provide broad categories of employment classification.

### Exposure coding

Three researchers (RD, ED, and SS) were involved in the exposure coding process. All three have significant experience of tobacco control and specifically the measurement and prevalence of exposure to second-hand smoke, including occupational exposure.

Each researcher scored each code on likelihood, frequency, and intensity of exposure. Full coding classifications are presented in Table 1. Where an assessor rated an SOC code with a likelihood rating of ‘0’ (<10% of workers likely to be exposed to SHS) no further rating for frequency and intensity was required, while those rated ‘1’ (10% or more of workers likely to be exposed to SHS) were rated for frequency and intensity of exposure.

**Table 1.** Exposure rating scheme.

Categorical rating	Likelihood of exposure	Frequency of exposure	Intensity of exposure
0	<10% of jobholders exposed at work	Never	None
1	10% or more of jobholders exposed at work	Less than once per week	Outdoor or passing exposure
2	—	At least once per week but less than daily	Indoors in a well-ventilated space
3	—	Daily	Indoors in a poorly ventilated space
4	—	Daily and more than 1 h of exposure per day	—

Rating was conducted independently by each researcher in the first instance. Following this process, each researcher’s ratings were compared statistically. Researchers engaged in consensus discussion about conflicting ratings, then conducted a second round of rating. After this process, any remaining conflicting ratings were assigned the value given by two researchers or, where only two researchers had rated the parameter, the higher value was taken.

### Exposure parameters

Occupational exposure to SHS was defined as any exposure to SHS at work, including SHS related to customers or colleagues smoking during the work day either outside or inside the premises.

### Workforce size estimates

Estimated numbers of jobholders were provided using the UK Office for National Statistics official labour market statistics (Nomis) standard reports data (Office for National Statistics, 2019) which provides an estimate for the number of people holding a job classified in each four-digit SOC code. These data were extracted in November 2020. These classifications were available only by SOC2010 codes, whereas this JEM was developed using SOC2020. To make compatible with our JEM (developed using SOC2020), Nomis job classification data were grouped by their four-digit SOC2010 code and this was subsequently recoded to their equivalent SOC2020 code.

### Statistical analysis

Inter-rater agreement was calculated using Fleiss’ *kappa* following the initial round of rating and the second round. Inter-rater agreement was only calculated for likelihood of exposure, to avoid artificially reducing apparent agreement (since frequency and intensity of exposure will, by definition, be zero when no exposure exists).

To provide a compound measure of exposure severity between different four-digit code occupations, the agreed ratings of likelihood, frequency, and intensity of exposure were multiplied. Means of this measure by two-digit code were compared with National Statistics Socio-Economic Classification (NS-SEC) analytic classes (Office for National Statistics, 2021) to assess the socioeconomic status of workers exposed and not exposed to SHS. NS-SEC analytic classes provide an overview of employment conditions and labour relations among different occupational groups.

Statistical analysis was performed in R v4.0.3 (R Core Team, 2020) and Microsoft Excel.

## Results

### Inter-rater agreement

First-round inter-rater agreement was moderate (Landis and Koch, 1977), with Fleiss’ *kappa* = 0.42 (with raters disagreeing on the likelihood of exposure in 111 of 412 codes). Following discussion and reflection, second-round agreement increased to Fleiss’ *kappa* = 0.66 (substantial agreement) with disagreement on the likelihood of exposure in a total of 67 codes. Disagreement between at least two raters at second round included the likelihood of exposure for specific categories of health professionals (SOC two-digit code 22), with disagreement on 10/24 four-digit codes, agricultural workers (SOC two-digit code 51) with disagreement on 5/5 four-digit codes and elementary trades (SOC two-digit code 91) with disagreement on 4/8 four-digit codes.

### Likelihood of exposure by occupation

Overall, 84 of 412 four-digit SOC codes (20.4%) were considered likely to have at least 10% of workers experiencing some degree of exposure to SHS during their duties.

Occupations classified as ‘skilled agricultural trades’ and ‘skilled construction and building trades’ were considered by raters most likely to be exposed to SHS, with 5/5 four-digit codes (100%) in the former category and 11/12 (92%) four-digit codes in the latter rated likely. Elementary administration and service occupations (16/26; 62%), elementary trades and related occupations (4/8; 50%) and caring personal service occupations (7/16; 44%) were also likely to be exposed (Supplementary Table S1, available at *Annals of Work Exposures and Health* online). In contrast, occupations likely to take place primarily in offices, such as administrative occupations (0/20) or science, research, engineering, and technology professionals (0/28), were rated unlikely to feature exposure to SHS.

A total of 10.4 million workers in the UK are employed in jobs (representing 22.6% of jobs held) identified as likely to experience some degree of exposure to SHS at work (Table 2). Our threshold of ‘10% of the workers within that code being exposed’ thus equates to at least 1.04 million workers exposed to SHS at work.

**Table 2.** Estimated number of UK jobholders by frequency and intensity of potential SHS exposure at work.

Frequency	Intensity				
	0 (never)	1 (outdoor or passing exposure)	2 (indoors in a well-ventilated space)	3 (indoors in a poorly ventilated space)	Total
0 (never)	35 729 200	—	—	—	35 729 200
1 (less than once per week)	—	—	17 900	1 587 800	1 605 700
2 (at least once per week but less than daily)	—	83 500	408 800	2 706 700	3 199 000
3 (daily)	—	3 037 700	904 600	789 000	4 731 300
4 (daily and more than 1 h of exposure per day)	—	—	—	884 700	884 700
Total	35 729 200	3 121 200	1 331 300	5 968 200	46 149 900

### Frequency and intensity of exposure by occupation

Occupations where the intensity of exposure to SHS was classified as high included those where work involved home visits, including healthcare providers and technicians. Agricultural occupations, by contrast, were generally assessed to have low intensity of exposure due to working primarily outdoors, despite high-frequency exposure.

### Overall exposure severity by exposure

Three four-digit code occupations had the highest possible level of exposure, according to the compound measure of severity (likelihood × frequency × intensity of exposure). These three occupations—nannies and au pairs (SOC code 6116), care workers and home carers (6135), and care escorts (6137)—all work regularly in other people’s homes. Around 884 700 jobholders (1.9% of the workforce) were estimated to fall into this category (Table 2). Another 789 000 people work in occupations (1.7%) where exposure to SHS occurs daily in an indoor space, representing 13% of all workers in job codes where work SHS exposure likely occurs indoors (Office for National Statistics, 2020). The overall mean compound measure of severity was 1.0 across all codes (range 0–12).

Three NS-SEC analytic categories had mean compound measures of severity above 1: small employers and own account workers (category 4, mean compound measure: 2.27), semi-routine occupations (category 6, mean compound measure 1.08), and routine occupations (category 7, mean compound measure 1.7). Category 4 workers are frequently self-employed, and those with high compound measures included plumbers, carpenters, bed and breakfast owner/operators, and others reflecting visits into homes as part of their work. Potentially severely exposed category 6 workers included care workers, with nannies and care escorts the most highly exposed occupations in category 7.

## Discussion

Over the course of this study, we assessed that approximately one in five four-digit SOC codes are likely to include a substantial group of workers who are regularly exposed to SHS in the course of their duties. This represents around 13.5% of job titles listed by the ONS (Semple *et al.*, 2019). We estimate that over 10 million workers (more than a fifth of the UK workforce) work in jobs where they may be exposed to SHS. This accords with other measures of SHS exposure, such as research finding that 18% of Scottish non-smoking adults have measurable levels of salivary cotinine on any given day (Avila-Tang *et al.*, 2013).

Occupations rated more likely to feature exposure to SHS frequently involved home visits (such as in the case of care workers). NS-SEC analytic groups 4, 6, and 7 included more potentially exposed occupations than higher-socioeconomic status (SES) categories. This study suggests that SHS exposure at work is also more common for people in lower-SES groups, further highlighting that the health harm of tobacco is principally focussed on the poorest people in the UK.

Our SHS-JEM stresses how smoking in the home is not only a health concern for people living there, but also for those whose work requires them to enter other people’s homes and highlights priority occupations for further assessment of SHS exposure and risk. Home care workers and similar in-home occupations such as nannies and au pairs were assessed as particularly heavily exposed to SHS in this study, and should be a focus of future research into occupational SHS exposure.

This paper outlines a methodology for creating a JEM for SHS exposure which could be repeated in other countries, including those without comprehensive smoke-free places legislation, to assess relative risks, progress, and harm from SHS by occupation and identify occupations for intervention development. Required data sources for use in other countries would include a comprehensive occupational classification system (equivalent to SOC) linked to a socioeconomic classification system (equivalent to NS-SEC). The granularity of these classification systems would affect the results—for example, broader category systems (equivalent to two-digit SOC codes) would make reduce the specificity of the resulting JEM, potentially missing higher exposure occupations, while more granular classification systems would increase the work involved in the process. Data sources used must be robust and comprehensive in order to provide a full understanding of occupational SHS exposure in a setting. Involving occupational hygienists with an interest in tobacco control and a good knowledge of local and national working practices would be advisable.

### Limitations

This JEM represents the views and knowledge of three researchers with significant scientific experience in occupational exposure to SHS, but it has not been possible to validate it directly through objective measurement of exposure. A programme of validation could include both objective measurement of SHS exposure in a range of occupations as well as survey components. When well designed, surveys can provide reliable and useful information on SHS exposure (Avila-Tang *et al.*, 2013).

It should also be noted that some workers may not be classified within the SOC scheme, including those in illegal or semi-legal industries (such as sex work). This limitation in the underlying source of data means that some workers may not be included in the JEM as developed.

## Conclusions

Well over a decade after smoke-free workplace legislation was introduced across the UK, more than a million working people continue to be occupationally exposed to SHS. This exposure is greatest among those who work inside others’ homes, including care workers, and among other low-SES workers. Addressing occupational exposure to SHS should be a health priority.

## Supplementary Material

wxab019_suppl_Supplementary_Table_1
