# Supplementary material for: Occupational Exposure to Second-Hand Tobacco Smoke: Development of a Job Exposure Matrix
Source: Ann Work Expo Health. 2021 Apr 3;65(9):1133–8. doi: 10.1093/annweh/wxab019 (PMC8675403; doi:10.1093/annweh/wxab019)
Supplement: wxab019_suppl_Supplementary_Table_1 [file wxab019_suppl_supplementary_table_1.docx]

Supplementary material

# Occupational exposure to second-hand tobacco smoke: development of a job exposure matrix

Ruaraidh Dobson^1*^, Evangelia Demou^2^, Sean Semple^1^

^1^ Institute for Social Marketing and Health, University of Stirling, UK
^2^ MRC-CSO Social and Public Health Sciences Unit, University of Glasgow, UK

^*^ Corresponding author ([r.p.dobson@stir.ac.uk](mailto:r.p.dobson@stir.ac.uk))

Supplementary Table 1 – Assessed exposure by two-digit code

| **SOC2020 Group Title** | **Two-digit code** | **Exposed four-digit codes / total four-digit codes (percentage)** | **Mean compound measure of exposure among four-digit codes (range)** |
| --- | --- | --- | --- |
| Corporate managers and directors | 11 | 0/20 (0%) | 0 (0-0) |
| Other managers and proprietors | 12 | 5/22 (23%) | 0.7 (0-4) |
| Science, research, engineering and technology professionals | 21 | 0/28 (0%) | 0 (0-0) |
| Health professionals | 22 | 7/24 (29%) | 1.6 (0-6) |
| Teaching and other educational professionals | 23 | 0/13 (0%) | 0 (0-0) |
| Business, media and public service professionals | 24 | 6/32 (19%) | 1 (0-9) |
| Science, engineering and technology associate professionals | 31 | 1/11 (9%) | 0.3 (0-3) |
| Health and social care associate professionals | 32 | 4/13 (31%) | 1.8 (0-6) |
| Protective service occupations | 33 | 2/5 (40%) | 1.2 (0-3) |
| Culture, media and sports occupations | 34 | 0/13 (0%) | 0 (0-0) |
| Business and public service associate professionals | 35 | 1/26 (4%) | 0.1 (0-3) |
| Administrative occupations | 41 | 0/20 (0%) | 0 (0-0) |
| Secretarial and related occupations | 42 | 0/7 (0%) | 0 (0-0) |
| Skilled agricultural and related trades | 51 | 5/5 (100%) | 3.6 (3-6) |
| Skilled metal, electrical and electronic trades | 52 | 9/23 (39%) | 1.8 (0-6) |
| Skilled construction and building trades | 53 | 11/12 (92%) | 3.8 (0-6) |
| Textiles, printing and other skilled trades | 54 | 1/17 (6%) | 0.4 (0-6) |
| Caring personal service occupations | 61 | 7/16 (44%) | 3.9 (0-12) |
| Leisure, travel and related personal service occupations | 62 | 3/11 (27%) | 1.6 (0-6) |
| Community and civil enforcement occupations | 63 | 0/2 (0%) | 0 (0-0) |
| Sales occupations | 71 | 1/13 (8%) | 0.7 (0-9) |
| Customer service occupations | 72 | 0/6 (0%) | 0 (0-0) |
| Process, plant and machine operatives | 81 | 0/25 (0%) | 0 (0-0) |
| Transport and mobile machine drivers and operatives | 82 | 1/14 (7%) | 0.4 (0-6) |
| Elementary trades and related occupations | 91 | 4/8 (50%) | 2 (0-6) |
| Elementary administration and service occupations | 92 | 16/26 (62%) | 2.3 (0-9) |
